# Supplementary material for: Origamic metal-organic framework toward mechanical metamaterial
Source: Nat Commun. 2023 Dec 1;14:7938. doi: 10.1038/s41467-023-43647-8 (PMC10692132; doi:10.1038/s41467-023-43647-8)
Supplement: Supplementary file 1 — Supplementary Information [file 41467_2023_43647_MOESM1_ESM.pdf]

Supplementary Information

Origamic Metal-Organic Framework toward Mechanical Metamaterial

Jin et al

Supplementary Methods

Ligand Synthesis

The 5, 10, 15, 20-tetrakis [4-carboxymethyleneoxyphenyl] porphyrin (TCMOPP) was synthesized by the literature.[1,2] The synthesis for the porphyrin linker consists of three steps.

Synthesis of 4-carboethoxymethyleneoxybenzaldehyde: 4-hydroxybenzaldehyde (12.2 g, 100 mmol) and ethyl bromoacetate (10.6 mL, 100 mmol) were dissolved in 100 mL of acetone. The solution added to anhydrous potassium carbonate (13.8 g, 100 mmol) was reacted for 8 hours at 70 °C using reflux. After the reaction, all solution was removed by using a rotary evaporator and extracted with $H_2O/CHCl_3$. The solvent of the separated organic phase was removed, and the crude product was obtained from a mixture of 0.5 % MeOH in $CHCl_3$. A solid white product was obtained. ^{1}H NMR (400 MHz, $CDCl_3$) δ 9.87 (s, 1H), 7.82 (d, 2H), 6.97 (d, 2H), 4.68 (s, 2H), 4.26 (q, 2H), 1.27 (t, 3H).

Synthesis of TEMOPP (5, 10, 15, 20-tetrakis [4-carboethoxymethyleneoxyphenyl] porphyrin): 4-carboethoxymethyleneoxybenzaldehyde (4.2 g, 20.19 mmol) and pyrrole (1.4 mL, 20.27 mmol) were dissolved in 20 mL of nitrobenzene and 40 mL of propionic acid. The solution was heated at 170 °C for 1 hour and then stored for 24 hours at room temperature. After then, the solvent of the mixture was removed except for a small volume and the silica gel was added to the mixture. The powder was loaded on the silica of the chromatography column. The crude product was obtained with 2 % acetone in $CHCl_3$. The violet crude product was recrystallized with MeOH and $CHCl_3$ at low temperatures. The purple powder was filtered and dried in an oven. ^{1}H NMR (400 MHz, $CDCl_3$) δ 8.84 (s, 8H), 8.12 (d, 8H), 7.29 (d, 8H), 4.92 (s, 8H), 4.41 (q, 8H), 1.41 (t, 12H), -2.78 (s, 2H).

Synthesis of TCMOPP (5, 10, 15, 20-tetrakis [4-carboxymethyleneoxyphenyl] porphyrin): TEMOPP (0.2 g, 0.195 mmol) was refluxed with sodium hydroxide (67 mg, 1.675 mmol) and H_2O of 2 mL in MeOH of 20 mL for 4 hours. After then, the solvent was removed by using a rotary evaporator. 4 mL of 0.5 N HCl solution was added to a crude product. After protonation, the color of the precipitate is green. The product was filtered, washed with water, and dried. The green product was dissolved in 600 μL of pyridine for neutralization of porphyrin and pyridine was removed by a rotary evaporator. Then, the final product was washed with water, filtered, and dried under a vacuum (94 %). ^{1}H NMR (400 MHz, DMSO-D6) δ 8.82 (s, 8H), 8.10 (d, 8H), 7.33 (d, 8H), 4.94 (s, 8H), -2.92 (s, 2H).

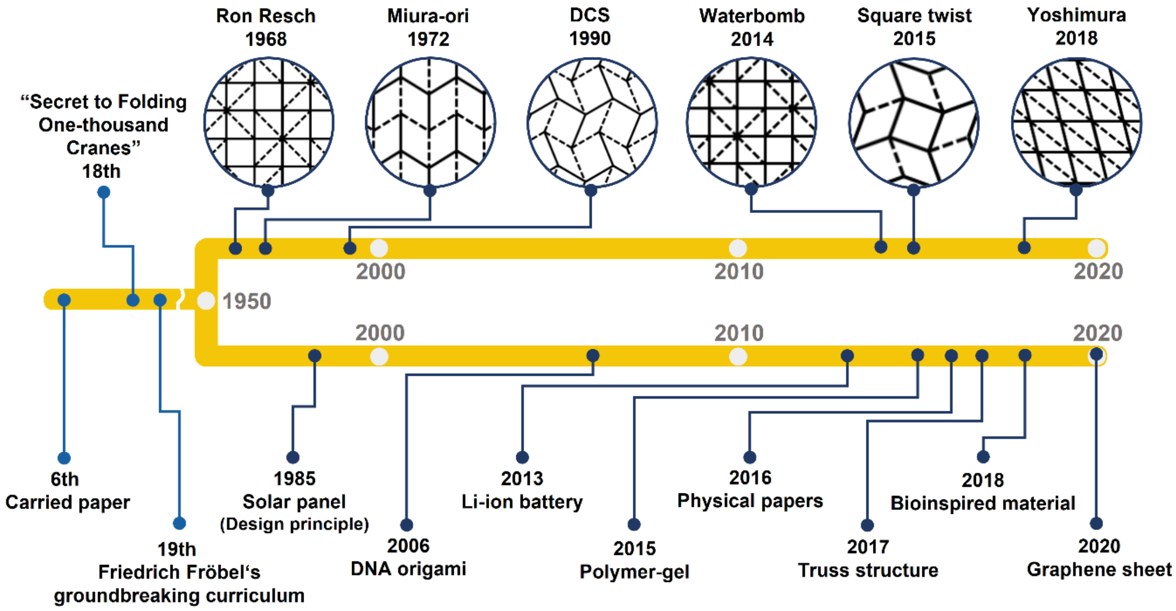

Supplementary Figure 1. A brief history of origami tessellations and their applications.

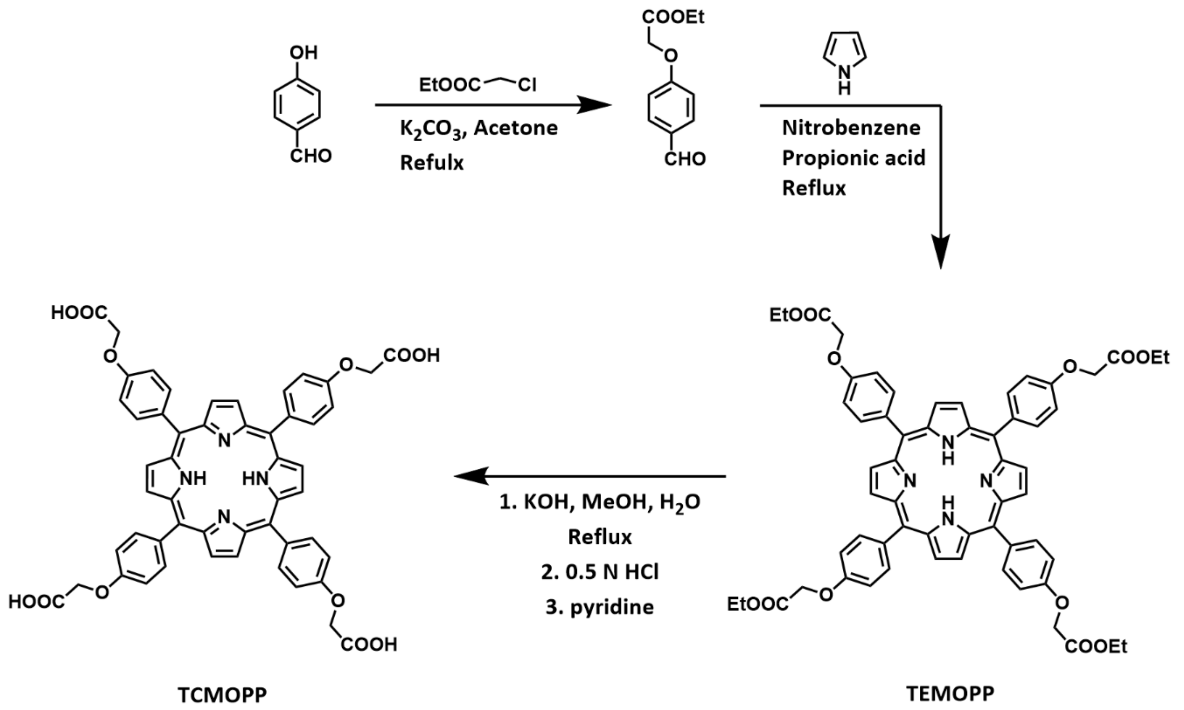

Supplementary Figure 2. The synthetic procedure of TCMOPP. The synthesis for TCMOPP consists of three steps.

Supplementary Note 1. Computational Details

Potential energy surface. Potential energy surface (PES) calculations were done with density functional theory using the hybrid functional B3LYP[3–6], as implemented in the Gaussian 16 package[7]. Empirical dispersion corrections D3 by Grimme and coworkers were added, with Becke-Johnson damping.[8] For the basis set, cc-pVDZ was used.[9] The structure of the isolated aryloxy group was specified with internal coordinates including α and φ. The scan was made adiabatically in increments of Δα = Δφ = 1° within the ranges.

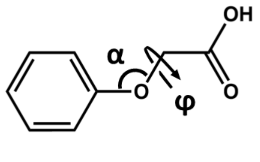

Phenoxyacetic acid

Elastic constants. Quantum mechanical calculations were performed based on density-functional theory (DFT) using the VASP program.[10] For DFT calculation, Perdew-Burke-Ernzerhof exchange-correlation functional[11] was used with the plane wave cutoff of 500 eV. Further, DFT-D3 dispersion correction with Becke-Johnson damping function was used.[12] The initial molecular configuration was constructed from the experimental X-ray crystal structure which contains 1,208 atoms in a unit cell with a volume of 1.3×10^4 Å^3. First, geometries as well as lattice parameters were optimized at Γ-point since the unit cell is reasonably large. The optimized lattice parameters with DFT are $a = 21.158$ Å, $b = 23.866$ Å, $c = 27.677$ Å, $\alpha = 98.622°$, $\beta = 104.977°$, and $\gamma = 103.696°$, implying similar crystal structure with the experimental X-ray crystal structure (Supplementary Table 1). In the cartesian coordinate, the optimized cell parameters are represented as $\boldsymbol{a} = (21.16, 0.0, 0.0)$, $\boldsymbol{b} = (-5.65, 23.19, 0.0)$, and $\boldsymbol{c} = (-7.15, -6.01, 26.05)$ in Å.

In addition, elastic constants were obtained by calculating total electronic energies to external distortions from the ElaStic program[13]. Using Voigt notation, the relation between Lagrangian strains η_i and stresses τ_i can be written as

$$\tau_i = \sum_{j=1}^{6} C_{ij}\eta_i$$

where C_{ij} is the stiffness tensor of material properties. Triclinic materials have 21 independent elements: C_{11}, C_{12}, C_{13}, ⋯, C_{66} and 21 independent distortions, D_i (i = 1, ⋯, 21) to lattice vectors were applied to determine these elements. Detailed information about the distortion and the energies of a strained crystal

can be found in the literature.[13] For the DFT method, 17 points for each distortion were used and the maximum Lagrangian strain is 0.04. Further, second order of elastic constants and energy choose as a method of calculation for the evaluation of elastic constants. From the above distortions D_i, C_{ij}'s were obtained based on polynomial fittings to the energies of a strained crystal. Polynomial functions are well fitted to the data points. There are the calculated stiffness components (C_{ij}'s) in the GPa unit,

$$\begin{pmatrix} C_{11} & C_{12} & C_{13} & C_{14} & C_{15} & C_{16} \\ C_{21} & C_{22} & C_{23} & C_{24} & C_{25} & C_{26} \\ C_{31} & C_{32} & C_{33} & C_{34} & C_{35} & C_{36} \\ C_{41} & C_{42} & C_{43} & C_{44} & C_{45} & C_{46} \\ C_{51} & C_{52} & C_{53} & C_{54} & C_{55} & C_{56} \\ C_{61} & C_{62} & C_{63} & C_{64} & C_{65} & C_{66} \end{pmatrix} = \begin{pmatrix} 15.4 & 7.0 & 4.9 & 2.0 & 1.2 & 3.7 \\ 0 & 10.8 & 6.8 & -0.2 & 0.3 & 3.2 \\ 0 & 0 & 13.0 & 0.2 & 1.3 & 1.9 \\ 0 & 0 & 0 & 5.7 & 0.4 & -0.0 \\ 0 & 0 & 0 & 0 & 4.6 & 1.9 \\ 0 & 0 & 0 & 0 & 0 & 4.3 \end{pmatrix}$$

and compliance components (S_{ij}'s) in 1/GPa unit

$$\begin{pmatrix} S_{11} & S_{12} & S_{13} & S_{14} & S_{15} & S_{16} \\ S_{21} & S_{22} & S_{23} & S_{24} & S_{25} & S_{26} \\ S_{31} & S_{32} & S_{33} & S_{34} & S_{35} & S_{36} \\ S_{41} & S_{42} & S_{43} & S_{44} & S_{45} & S_{46} \\ S_{51} & S_{52} & S_{53} & S_{54} & S_{55} & S_{56} \\ S_{61} & S_{62} & S_{63} & S_{64} & S_{65} & S_{66} \end{pmatrix} = \begin{pmatrix} 0.109 & -0.052 & -0.005 & -0.040 & 0.003 & -0.055 \\ 0 & 0.201 & -0.078 & 0.024 & 0.060 & -0.096 \\ 0 & 0 & 0.120 & -0.003 & -0.037 & 0.025 \\ 0 & 0 & 0 & 0.191 & -0.017 & 0.027 \\ 0 & 0 & 0 & 0 & 0.289 & -0.161 \\ 0 & 0 & 0 & 0 & 0 & 0.413 \end{pmatrix}$$

Furthermore, the stability of the crystal was checked by using the fact that the strain energy ($C_{ij}\eta_i\eta_j$) must be positive-definite, calculating the determinants of the elastic stiffness constants matrices. PPF-301 satisfies this condition showing the values from 15.4 to 5.71 $\times$ 10^4 with DFT calculation.

In addition, Young's modulus (E), Poisson's ratio (v), shear modulus (G), and bulk modulus (B) can be obtained in terms of C_{ij}'s or S_{ij}'s. For the calculation of bulk and shear moduli; Voigt and Reuss representations as

$$B_V = \frac{1}{9}[(C_{11} + C_{22} + C_{33}) + 2(C_{12} + C_{13} + C_{23})]$$

$$G_V = \frac{1}{15}[(C_{11} + C_{22} + C_{33}) - (C_{12} + C_{13} + C_{23}) + 3(C_{44} + C_{55} + C_{66})]$$

and

$$B_R = [(S_{11} + S_{22} + S_{33}) + 2(S_{12} + S_{13} + S_{23})]^{-1}$$

$$G_R = 15[4(S_{11} + S_{22} + S_{33}) - 4(S_{12} + S_{13} + S_{23}) + 3(S_{44} + S_{55} + S_{66})]^{-1}$$

Young's modulus and Poisson's ratio can be simply calculated from bulk (B = 1/2(B_V + B_R)) and shear (G = 1/2(G_V + G_R)) moduli using the assumption that the material is isotropic. There are two representations according to the values of bulk and shear moduli, and these are expressed by

$$E = \frac{9BG}{3B + G}$$

$$\nu = \frac{3B - 2G}{2(3B + G)}$$

With the calculated elastic stiffness constants, the spatial dependence of elastic moduli and Poisson's ratio were analyzed from the ELATE software.[14] Maximum and minimum values, as well as anisotropy (A) for Young's modulus, linear compressibility (β), shear modulus, and Poisson's ratio, were obtained. Linear compressibility is defined as a reciprocal number of the Bulk modulus; thus, it has a unit of 1/TPa. Anisotropy is defined as a maximal value of the elastic moduli divided by a minimal value. When the value becomes negative, the anisotropy is expressed to infinity.

Supplementary Note 2. Structural analysis and characterization of PPF-301

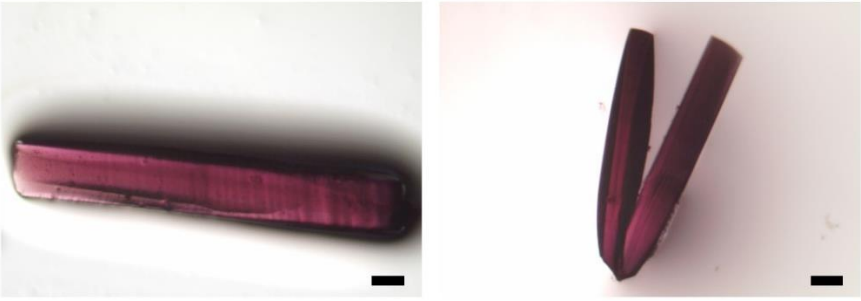

Supplementary Figure 3. Optical microscope images of PPF-301. The crystal exhibits a rectangular plate shape with a pale purple color (Scale bar: 0.1 mm).

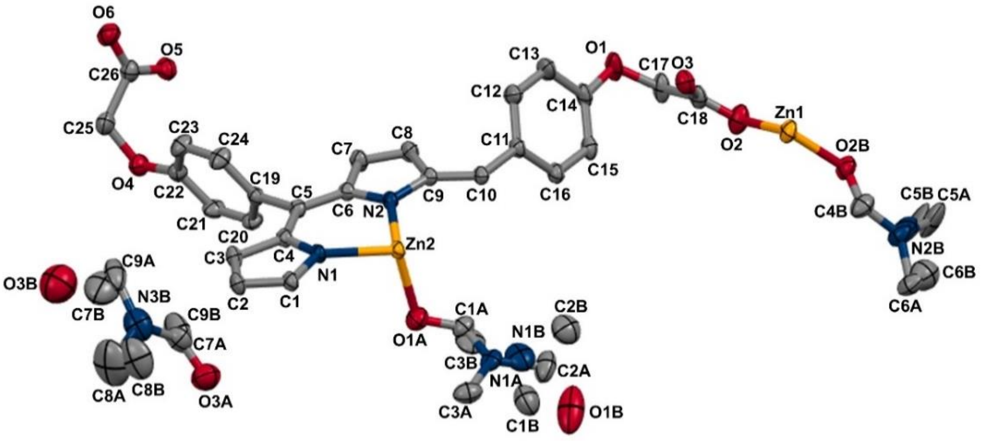

Supplementary Figure 4. The asymmetric unit of PPF-301 based on crystallographic data obtained at 100 K. Copper, carbon, nitrogen, and oxygen atoms are indicated in orange, gray, blue, and red, respectively. Hydrogen atoms have been omitted for clarity. (Displacement ellipsoids are shown at a 50 % probability).

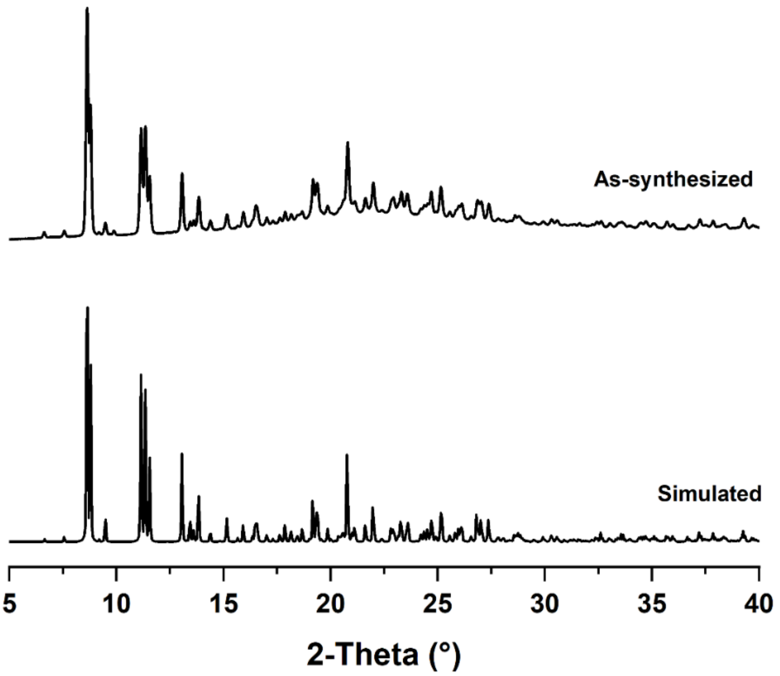

Supplementary Figure 5. Synchrotron powder X-ray diffraction data of the as-synthesized PPF-301. The simulated reflection data is compared with the experimental results.

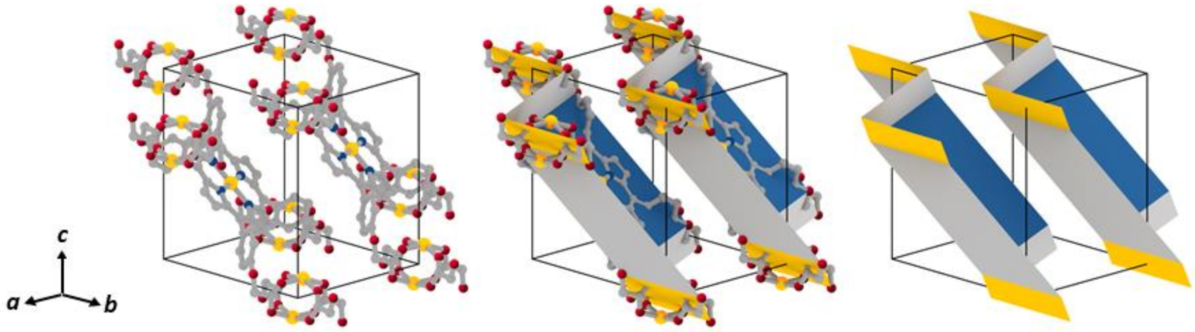

Supplementary Figure 6. The stacking of the sheets in a unitcell, embedded to $(\bar{1}11)$ plane.

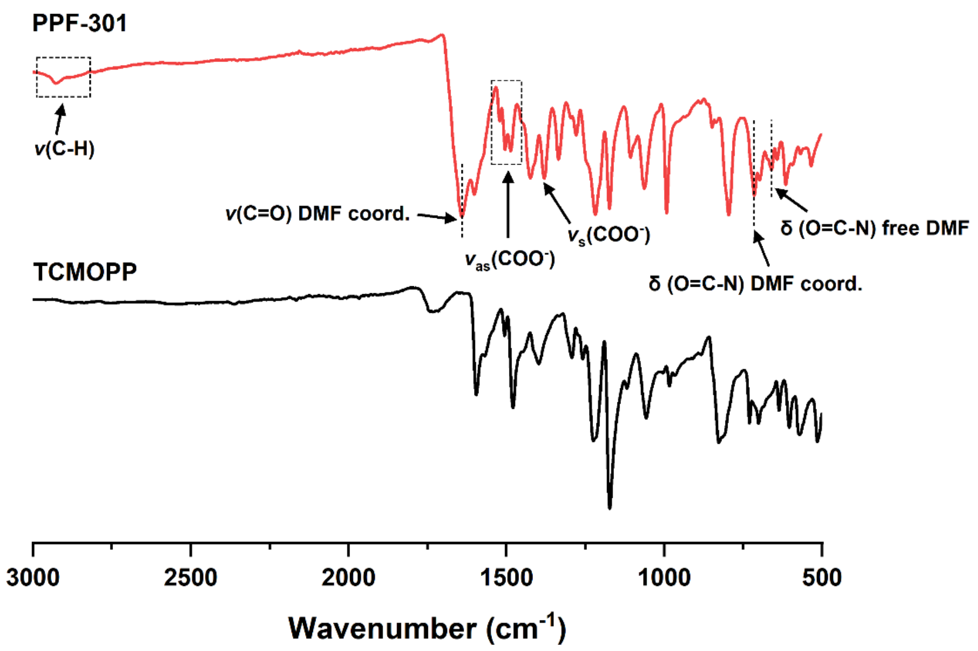

Supplementary Figure 7. FT-IR spectra of TCMOPP and PPF-301. The *v*(C=O) and *δ*(O=C-N) bands indicate the presence of coordinated DMF molecules in PPF-301.

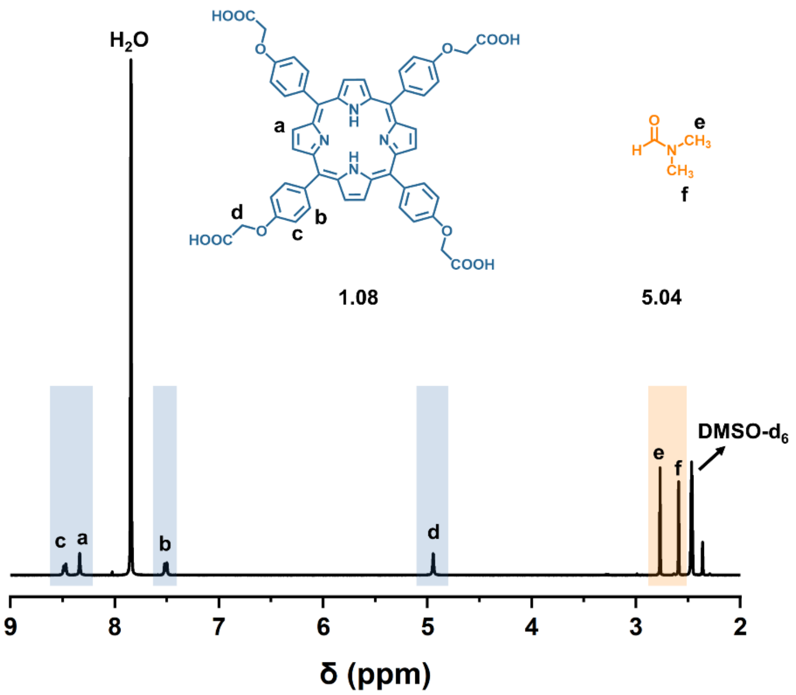

Supplementary Figure 8. ¹H NMR spectrum showing the trace of solvent content in PPF-301. The crystals were washed with the mother liquid, filtered, and then digested using 0.5 mL of DMSO-d_6 and 0.1 mL of dilute DCl (0.1 mL of 35 % DCl in D_2O in 0.5 mL DMSO-d_6). The ratio of TCMOPP to DMF was determined to be 1.08:5.04.

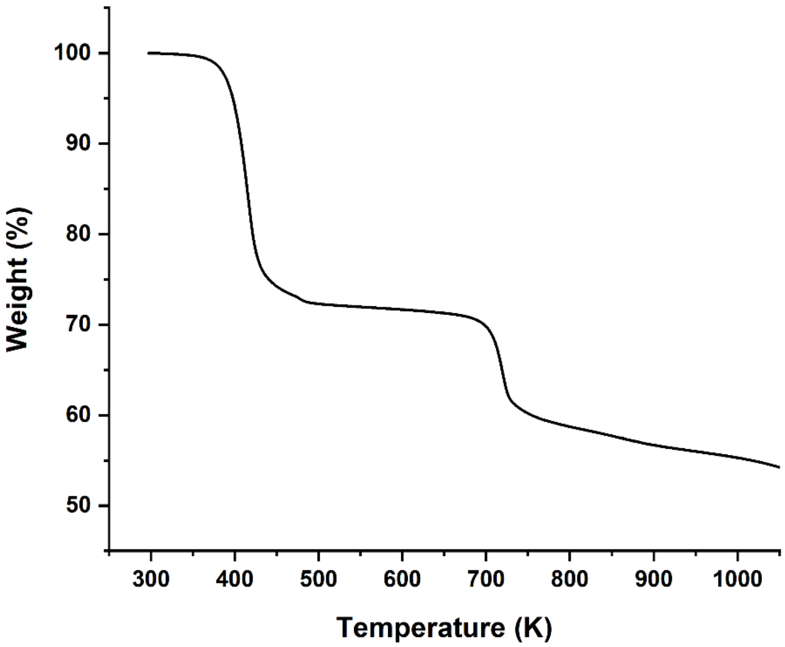

Supplementary Figure 9. Thermogravimetric analysis data for PPF-301. The initial weight loss (~27 %, 300–420 K) corresponds to solvent removal, followed by decomposition of the structure at around 700 K (~40 % weight loss).

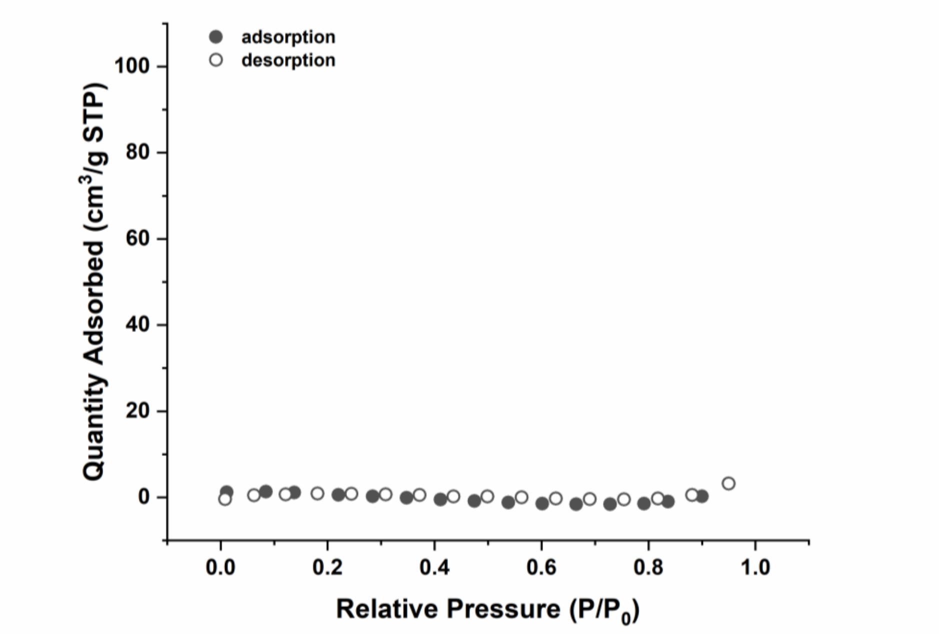

Supplementary Figure 10. N_2 isotherm of PPF-301 at 77 K.

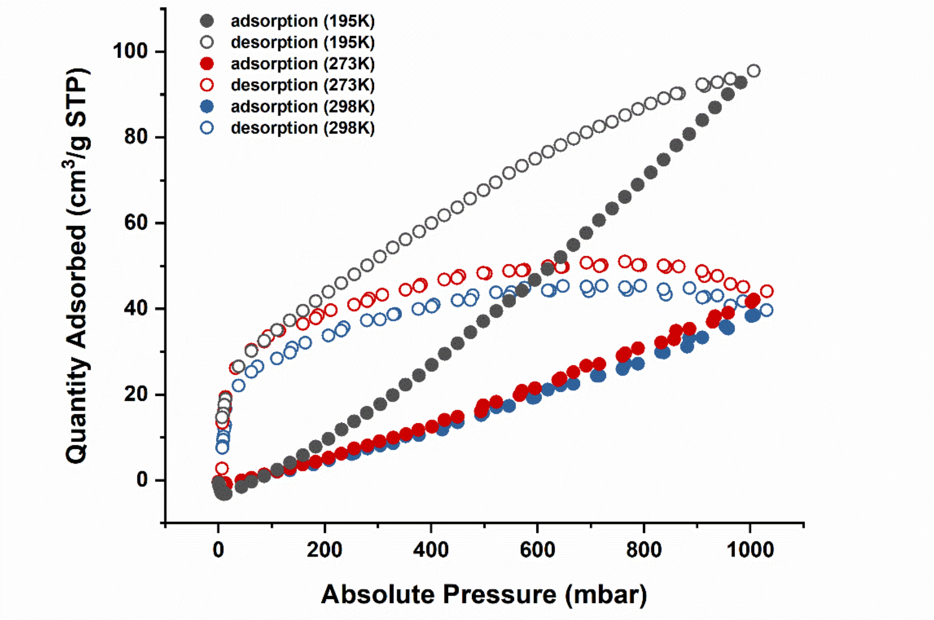

Supplementary Figure 11. CO_2 uptake of PPF-301 at 195, 273, and 298 K. The small amounts of CO_2 adsorbed 4.26, 1.96, and 1.77 mmol/g, respectively.

Supplementary Note 3. Temperature-dependent experiments

Supplementary Table 1. Temperature-dependent crystallographic data of PPF-301.

Complex	PPF-301_100	PPF-301_120	PPF-301_140	PPF-301_160
Formula	$C_{70}H_{74}N_{10}O_{18}Zn_3$	$C_{70}H_{74}N_{10}O_{18}Zn_3$	$C_{70}H_{74}N_{10}O_{18}Zn_3$	$C_{70}H_{74}N_{10}O_{18}Zn_3$
T (K)	100(2)	120(2)	140(2)	160(2)
Crystal system	Triclinic	Triclinic	Triclinic	Triclinic
Space group	*P-1*	*P-1*	*P-1*	*P-1*
a (Å)	10.855(2)	10.868(2)	10.885(2)	10.902(2)
b (Å)	12.536(3)	12.558(3)	12.583(3)	12.611(3)
c (Å)	14.026(3)	14.033(3)	14.038(3)	14.040(3)
V (Å^3)	1723.0(7)	1728.0(7)	1733.4(7)	1738.6(7)
α (°)	100.69(3)	100.68(3)	100.70(3)	100.72(3)
β (°)	101.29(3)	101.29(3)	101.26(3)	101.21(3)
γ (°)	107.50(3)	107.59(3)	107.72(3)	107.86(3)
Z	1	1	1	1
R1 [I > 2σ(I)]	0.0525	0.0526	0.0529	0.0534
wR2 [I > 2σ(I)]	0.1537	0.1586	0.1565	0.1577
R1 [all data]	0.0696	0.0701	0.0709	0.0727
wR2 [all data]	0.1609	0.1667	0.1646	0.1659
CCDC number	2122043	2122044	2122045	2122046

Complex	PPF-301_180	PPF-301_200	PPF-301_220	PPF-301_240
Formula	$C_{70}H_{74}N_{10}O_{18}Zn_3$	$C_{70}H_{74}N_{10}O_{18}Zn_3$	$C_{70}H_{74}N_{10}O_{18}Zn_3$	$C_{70}H_{74}N_{10}O_{18}Zn_3$
T (K)	180(2)	200(2)	220(2)	240(2)
Crystal system	Triclinic	Triclinic	Triclinic	Triclinic
Space group	*P-1*	*P-1*	*P-1*	*P-1*
a (Å)	10.921(2)	10.941(2)	10.964(2)	10.986(2)
b (Å)	12.640(3)	12.674(3)	12.707(3)	12.739(3)
c (Å)	14.041(3)	14.043(3)	14.047(3)	14.050(3)
V (Å^3)	1744.1(7)	1750.3(7)	1757.0(7)	1763.4(7)
α (°)	100.74(3)	100.78(3)	100.80(3)	100.81(3)
β (°)	101.16(3)	101.10(3)	101.05(3)	101.00(3)
γ (°)	108.02(3)	108.21(3)	108.39(3)	108.57(3)
Z	1	1	1	1
R1 [I > 2σ(I)]	0.0539	0.0533	0.0543	0.0547
wR2 [I > 2σ(I)]	0.1591	0.1563	0.1587	0.1586
R1 [all data]	0.0741	0.0746	0.0782	0.0796
wR2 [all data]	0.1670	0.1648	0.1679	0.1681
CCDC number	2122047	2122048	2122049	2122050

Complex	PPF-301_260	PPF-301_280	PPF-301_300	PPF-301_320
Formula	$C_{70}H_{74}N_{10}O_{18}Zn_3$	$C_{70}H_{74}N_{10}O_{18}Zn_3$	$C_{70}H_{74}N_{10}O_{18}Zn_3$	$C_{70}H_{74}N_{10}O_{18}Zn_3$
T (K)	260(2)	280(2)	300(2)	320(2)
Crystal system	Triclinic	Triclinic	Triclinic	Triclinic
Space group	*P-1*	*P-1*	*P-1*	*P-1*
a (Å)	11.010(2)	11.035(2)	11.061(2)	11.089(2)
b (Å)	12.773(3)	12.807(3)	12.842(3)	12.877(3)
c (Å)	14.055(3)	14.060(3)	14.065(3)	14.071(3)
V (Å^3)	1770.5(7)	1777.9(7)	1785.6(7)	1793.5(7)
α (°)	100.81(3)	100.80(3)	100.77(3)	100.74(3)
β (°)	100.97(3)	101.95(3)	100.93(3)	100.94(3)
γ (°)	108.75(3)	108.93(3)	109.11(3)	109.29(3)
Z	1	1	1	1
R1 [I > 2σ(I)]	0.0546	0.0552	0.0554	0.0565
wR2 [I > 2σ(I)]	0.1581	0.1592	0.1599	0.1628
R1 [all data]	0.0829	0.0867	0.0913	0.0960
wR2 [all data]	0.1693	0.1706	0.1728	0.1771
CCDC number	2122051	2122052	2122053	2122054

Complex	PPF-301_340	PPF-301_360	PPF-301_380
Formula	$C_{70}H_{74}N_{10}O_{18}Zn_3$	$C_{70}H_{74}N_{10}O_{18}Zn_3$	$C_{70}H_{74}N_{10}O_{18}Zn_3$
T (K)	340(2)	360(2)	380(2)
Crystal system	Triclinic	Triclinic	Triclinic
Space group	*P-1*	*P-1*	*P-1*
a (Å)	11.115(2)	11.142(2)	11.168(2)
b (Å)	12.904(3)	12.925(3)	12.952(3)
c (Å)	14.076(3)	14.083(3)	14.088(3)
V (Å^3)	1800.1(7)	1806.3(7)	1812.8(7)
α (°)	100.70(3)	100.65(3)	100.60(3)
β (°)	100.96(3)	101.01(3)	101.05(3)
γ (°)	109.45(3)	109.59(3)	109.74(3)
Z	1	1	1
R1 [I > 2σ(I)]	0.0569	0.0585	0.0619
wR2 [I > 2σ(I)]	0.1633	0.1692	0.1859
R1 [all data]	0.1004	0.1089	0.1069
wR2 [all data]	0.1779	0.1868	0.2042
CCDC number	2122055	2122056	2122057

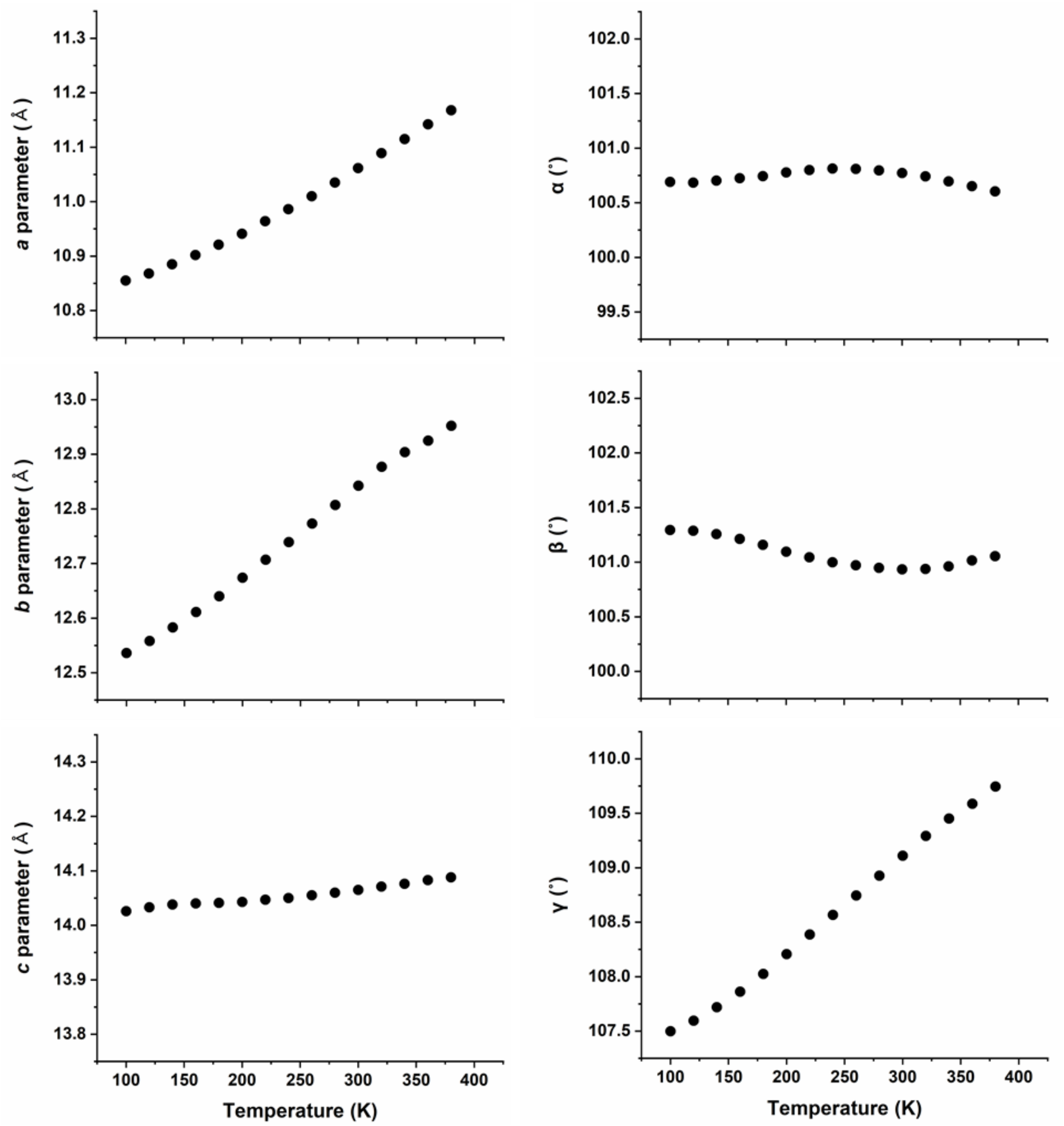

Supplementary Figure 12. Lattice parameters of PPF-301 at variable temperatures (The small error bar is represented in the graph).

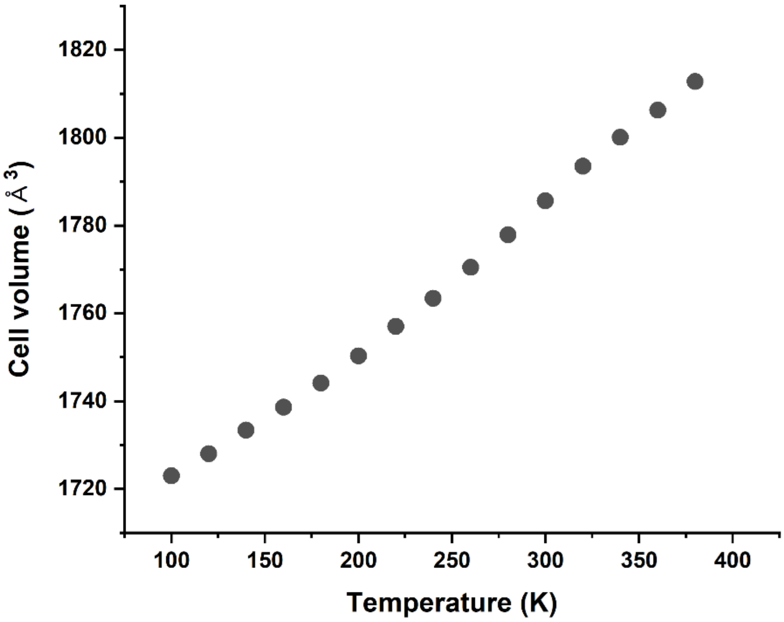

Supplementary Figure 13. Cell volume at variable temperature (The small error bar is represented in the graph).

Supplementary Table 2. The parameters including lengths, 2D area, interlayer spacing, and thickness at variable temperatures.

T	d_1	d_2	σ	*Area (S)*		*Interlayer*	d_3	
(K)	(Å)	(Å)	(°)	(Å²)	*(%)*	(Å)	(Å)	*(%)*
100	15.97	13.90	86.26	221.43	0.00	7.78	5.64	0.00
120	15.98	13.90	86.30	221.71	0.13	7.79	5.63	-0.17
140	16.00	13.91	86.35	222.03	0.27	7.81	5.62	-0.26
160	16.01	13.91	86.40	222.36	0.42	7.82	5.61	-0.48
180	16.03	13.92	86.45	222.68	0.56	7.83	5.60	-0.59
200	16.05	13.92	86.54	223.02	0.72	7.85	5.59	-0.72
220	16.08	13.92	86.59	223.43	0.90	7.86	5.59	-0.87
240	16.10	13.92	86.65	223.76	1.05	7.88	5.58	-1.06
260	16.12	13.93	86.70	224.16	1.24	7.90	5.56	-1.30
280	16.14	13.93	86.76	224.51	1.39	7.92	5.55	-1.50
300	16.16	13.94	86.80	224.89	1.56	7.94	5.54	-1.67
320	16.18	13.94	86.85	225.25	1.73	7.96	5.52	-1.98
340	16.19	13.95	86.87	225.51	1.84	7.98	5.51	-2.21
360	16.20	13.95	86.89	225.72	1.94	8.00	5.50	-2.38
380	16.21	13.96	86.92	225.95	2.04	8.02	5.49	-2.58

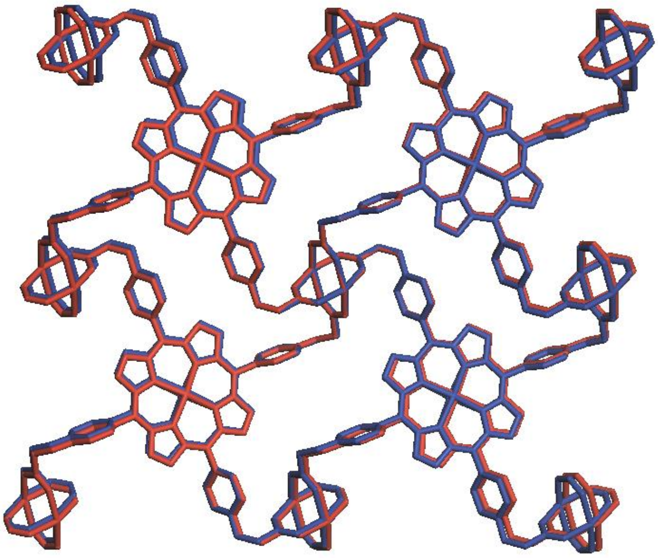

Supplementary Figure 14. Overlaid crystallographic structures at 100 K (blue color fragment) and 380 K (red color fragment).

Thermal expansion coefficient (α) of PPF-301

Supplementary Table 3. Principal coefficients of thermal expansion and corresponding principal axes, determined for PPF-301 (MK^{-1}, $10^{-6}\,K^{-1}$) in the range of 100–380 K.

Principal axis, *i*	α_i (MK^{-1})	Component of X_i along the crystallographic axes			Approximate axis
		**a**	**b**	**c**	
X_1	2.1(4)	0.6703	0.5718	0.4731	[122]
X_2	17(1)	-0.5970	-0.3233	0.7342	$[\bar{2}\bar{1}1]$
X_3	170(3)	0.6842	-0.7220	0.1024	$[1\bar{1}0]$
V	192(3)	-	-	-	-

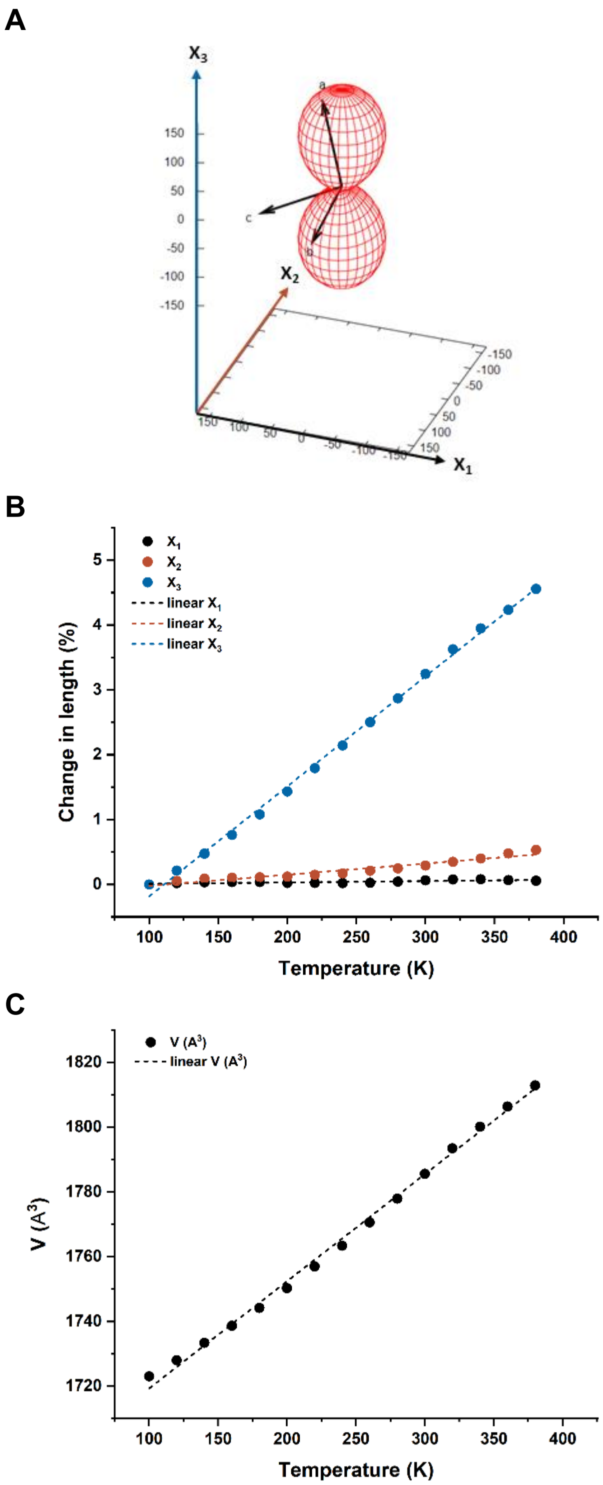

Supplementary Figure 15. Thermal expansion coefficients (α) of PPF-301. A The thermal expansion indicatrix (red positive) of PPF-301 along the principle orthogonal axis X_3. The fitting graph is the change of **B** lengths and **C** volume from 100 K to 380 K.

Supplementary Table 4. Thermal expansion coefficients (α) of 2D solid-state materials.

Compound	T (K)	max α (10^{-6} K^{-1})				Ref
		a (or X_1)	b (or X_2)	c (or X_3)	V	
PPF-301	100–380	2.1(4)	17(1)	170(3)	192(3)	This work
Zn2-1	80–293	12.09	14.08	49.32	78.78	15
Compound 1	100–350	-36(2)	2.5(9)	152(3)	119(2)	16
$[Ni(pba)_2]\cdot 2DMA$	112–300	153(2)	41(4)	-35(4)	159(3)	17
$[Co(pba)_2]\cdot 2DMA$	112–300	193(3)	64(4)	-76(3)	180(4)	17
$[Zn(pba)_2]\cdot 2DMA$	112–300	187(2)	89(3)	-84(3)	191(2)	17
$[Zn_{0.77}Cd_{0.23}(pba)_2]\cdot 2DMA$	112–300	188(4)	142(4)	-134(5)	195(5)	17
$[Cd(pba)_2]\cdot 2DMA$	112–300	226(2)	165(2)	-155(3)	233(3)	17
$Ni(CN_2)$	28–300	6.5(1)	-	61.8(3)	48.5(4)	18
$LiBeBO_3$	73–193	-3.31(13)	-176(08)	7.58(76)	-	19

Supplementary Note 4. Geometrical analysis of 2D sheets based on origami tessellation

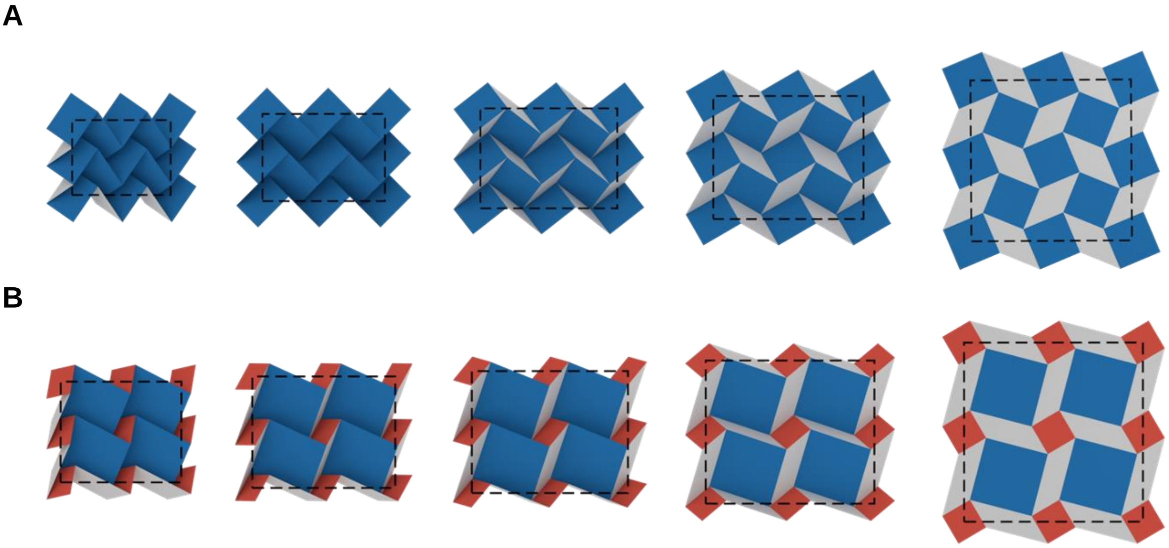

Supplementary Figure 16. Two types of DCS tessellations. A Same-sized squares and **B** Different-sized squares.

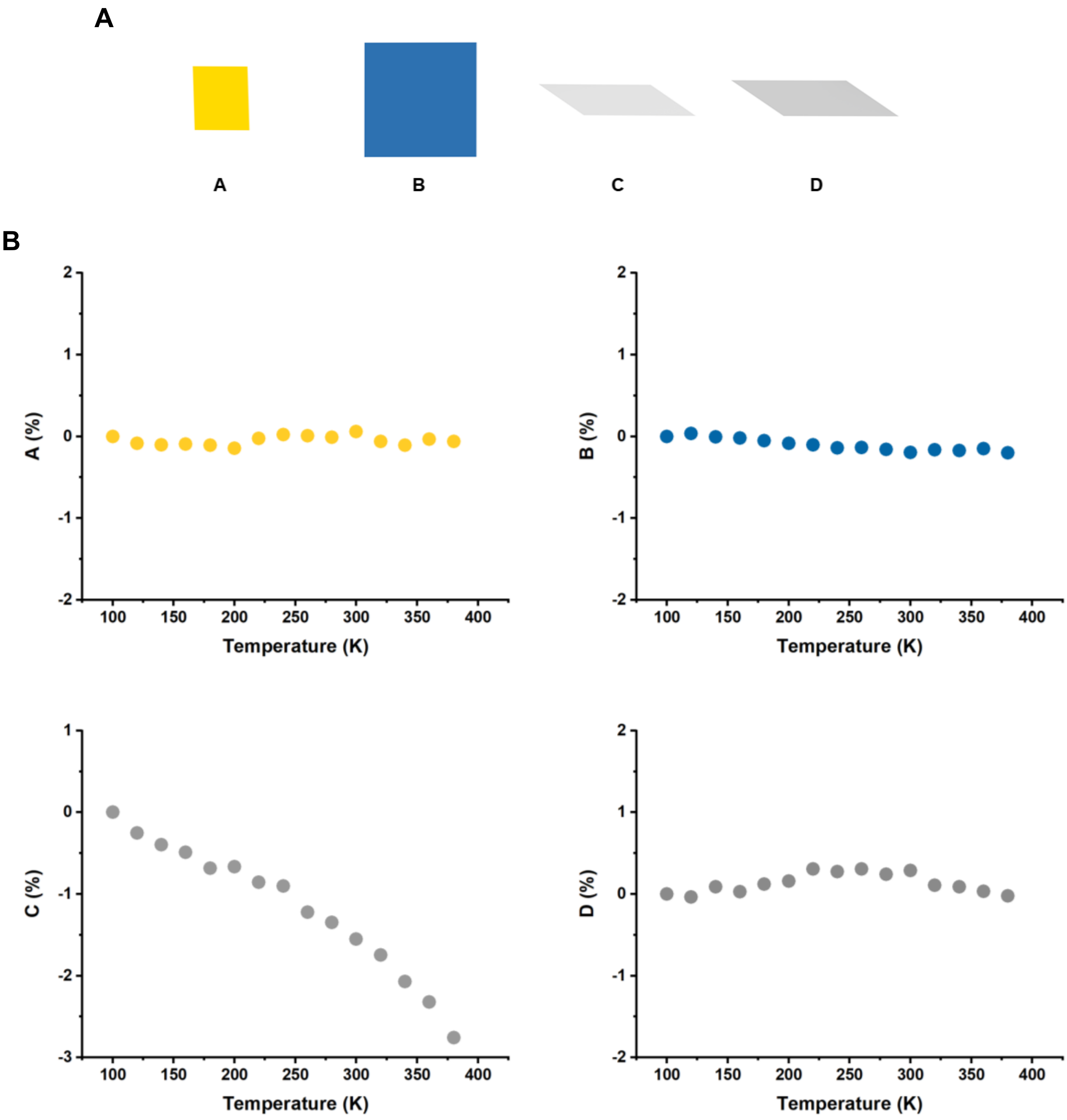

Supplementary Figure 17. Area changes of four types of tiles, filled in the 2D sheet at variable temperatures. **A** Four types of tiles of varying sizes. **B** Variation of area depending on temperatures. The A, B, and D are rarely changed, and the C was gradually reduced.

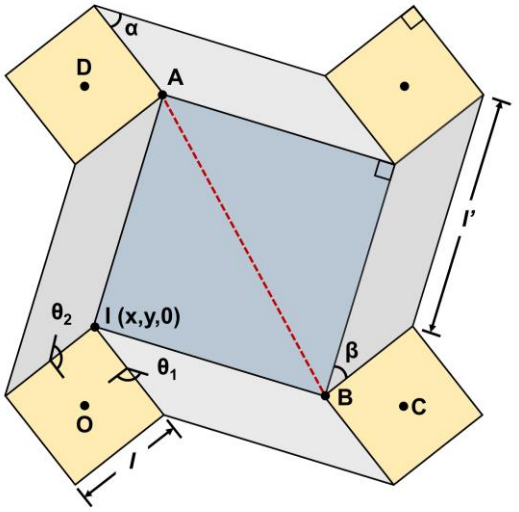

Supplementary Figure 18. Schematic representation in a folding model with four types of tiles. θ_1, θ_2: Folding angles, l_1, l_2, l_3, l_4: Edges of tiles, α, β: Actual angles of tiles.

(Constants: l = 6.751 Å, l' = 12.901 Å, α = 33.759°, β = 34.814°

Correlation between θ_1 and θ_2

$A\ (x + l' \cos\beta\,, y - l' \sin\beta \cos\theta_2\,, l' \sin\beta \sin\theta_2)$

$B(x - l' \sin\alpha \cos\theta_1\,, y - l' \cos\alpha\,, -l' \sin\alpha \sin\theta_1)$

$$\overline{AB} = \sqrt{l'^2 + l'^2 - 2l'^2}$$

$$\overrightarrow{BA} = (l' \cos\beta + l' \sin\alpha \cos\theta_1\,, l' \cos\alpha - l' \sin\beta \cos\theta_2\,, l' \sin\beta \sin\theta_2 + l' \sin\alpha \sin\theta_1)$$

$$\overline{AB}^2$$

$$= (l' \cos\beta + l' \sin\alpha \cos\theta_1)^2 + (l' \cos\alpha - l' \sin\beta \cos\theta_2)^2 + (l' \sin\beta \sin\theta_2 + l' \sin\alpha \sin\theta_1)^2$$
$$= l'^2 + l'^2 - 2l'^2$$

$$= -\sin\alpha \cos\beta \cos\theta_1 + \cos\alpha \sin\beta \cos\theta_2 - \sin\alpha \sin\beta \sin\theta_1 \sin\theta_2 = \cos\delta$$

Arrange in terms of $\sin\theta_2$

$$\cos\theta_2 = \frac{\sin\alpha \cos\beta \cos\theta_1}{\cos\alpha \sin\beta} + \frac{\sin\alpha \sin\beta \sin\theta_1 \sin\theta_2}{\cos\alpha \sin\beta}$$

$$\cos\theta_2 = \frac{\tan\alpha \cos\theta_1}{\tan\beta} + \tan\alpha \sin\theta_1 \sin\theta_2$$

$$1-\sin^2\theta_2 = \left(\frac{\tan\alpha\cos\theta_1}{\tan\beta}\right)^2 + (\tan\alpha\sin\theta_1\sin\theta_2)^2 + 2\left(\frac{\tan\alpha\cos\theta_1}{\tan\beta}\right)(\tan\alpha\sin\theta_1\sin\theta_2)$$

$$(\sin^2\theta_1\tan^2\alpha+1)\sin^2\theta_2 + \left(\frac{\tan^2\alpha\sin 2\theta_1}{\tan\beta}\right)\sin\theta_2 + \left[\left(\frac{\tan\alpha\cos\theta_1}{\tan\beta}\right)^2 - 1\right] = 0$$

Using Quadratic formula $(\sin\theta_2 > 0)$

$$\sin\theta_2 = \frac{-\left(\frac{\tan^2\alpha\sin 2\theta_1}{\tan\beta}\right) + \sqrt{\left(\frac{\tan^2\alpha\sin 2\theta_1}{\tan\beta}\right)^2 - 4(\sin^2\theta_1\tan^2\alpha+1)\left[\left(\frac{\tan\alpha\cos\theta_1}{\tan\beta}\right)^2 - 1\right]}}{2(\sin^2\theta_1\tan^2\alpha+1)}$$

Supplementary Equation (1).

$$\therefore \theta_2 = \sin^{-1}\left(\frac{-\left(\frac{\tan^2\alpha\sin 2\theta_1}{\tan\beta}\right) + \sqrt{\left(\frac{\tan^2\alpha\sin 2\theta_1}{\tan\beta}\right)^2 - 4(\sin^2\theta_1\tan^2\alpha+1)\left[\left(\frac{\tan\alpha\cos\theta_1}{\tan\beta}\right)^2 - 1\right]}}{2(\sin^2\theta_1\tan^2\alpha+1)}\right)$$

Correlation between d_1, d_2 and θ_1

$C\ (x + l'\cos\beta\,, y - l'\sin\beta\cos\theta_2\,, l'\sin\beta\sin\theta_2)$

$D\ (x - l'\sin\alpha\cos\theta_1\,, y - l'\cos\alpha\,, -l'\sin\alpha\sin\theta_1)$

$O\ (0, 0, 0)$

$\overrightarrow{CO} = (-l'\sin\alpha\cos\theta_1 - l'\cos\beta\,, l'\sin\beta\cos\theta_2 - l'\cos\alpha\,, -l'\sin\alpha\sin\theta_1 - l'\sin\beta\sin\theta_2)$

$\overrightarrow{DO} = (-l'\sin\alpha\cos\theta_1 - l'\cos\beta\,, l'\sin\beta\cos\theta_2 - l'\cos\alpha\,, -l'\sin\alpha\sin\theta_1 - l'\sin\beta\sin\theta_2)$

Supplementary Equation (2).

$$\therefore \overline{CO} = d_1 = \sqrt{(l - l'\sin\alpha\cos\theta_1)^2 + l'^2\cos^2\alpha + l'^2\sin^2\alpha\sin^2\theta_1}$$

$$\therefore \overline{DO} = d_2 = \sqrt{(l - l'\sin\beta\cos\theta_2)^2 + l'^2\cos^2\beta + l'^2\sin^2\beta\sin^2\theta_2}$$

Supplementary Table 5. The folding angles, θ_1 and θ_2 depending on temperatures.

T (K)	θ_1 (°)	θ_2 (°)
100	115.4	76.9
120	115.5	77.1
140	115.8	77.5
160	116.0	77.7
180	116.2	78.0
200	116.5	78.3
220	116.7	78.7
240	116.9	79.0
260	117.1	79.3
280	117.4	79.6
300	117.6	79.9
320	117.9	80.2
340	118.0	80.5
360	118.1	80.6
380	118.3	80.8

A

B

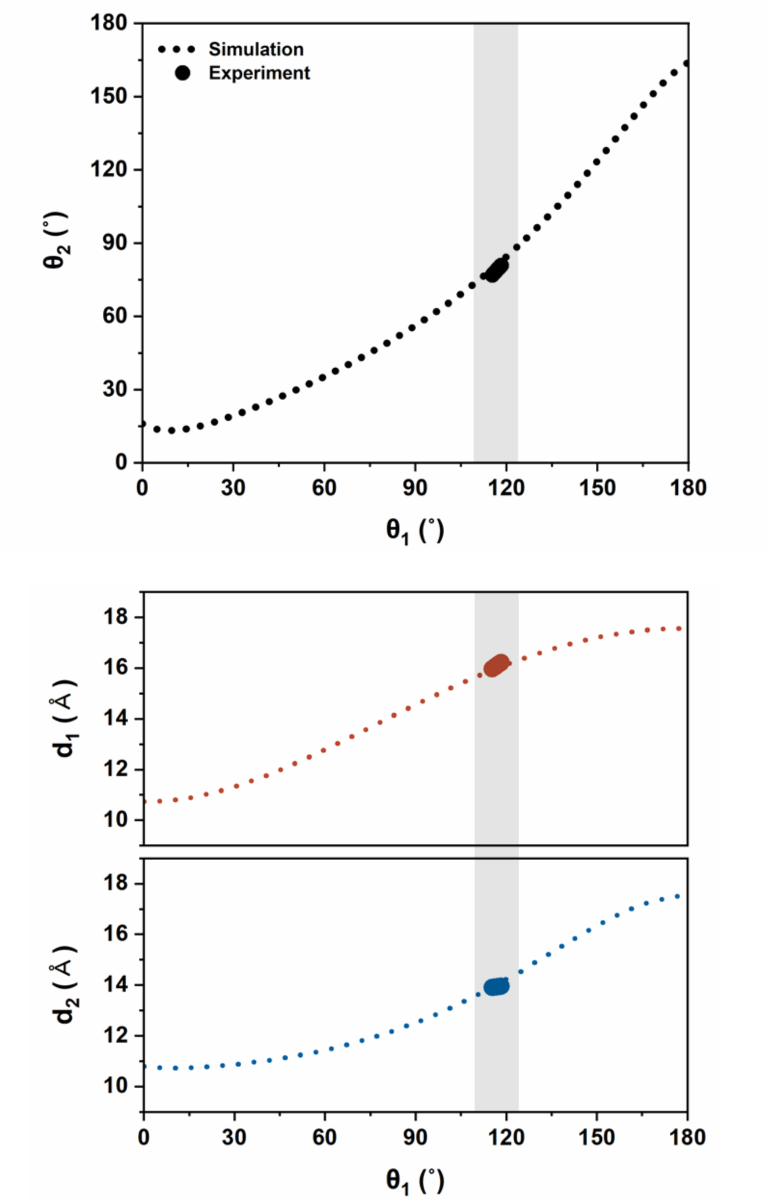

Supplementary Figure 19. A comparison between experiment and mathematical model. A Relationships between folding angles; θ_1 and θ_2. **B** Relationships between lengths (d_1 and d_2) and θ_1.

Supplementary Note 5. Origin of origami movement

Supplementary Table 6. Variation of dihedral angle (φ) and bond angle (α) of aryloxy group at 100 K and 380 K.

T (K)	φ_A (°)	α_A (°)	φ_B (°)	α_B (°)
100	±63.4	116.8	±71.2	116.5
380	±65.8	117.9	±72.1	117.2

Supplementary Note 6. Mechanical behavior of PPF-301

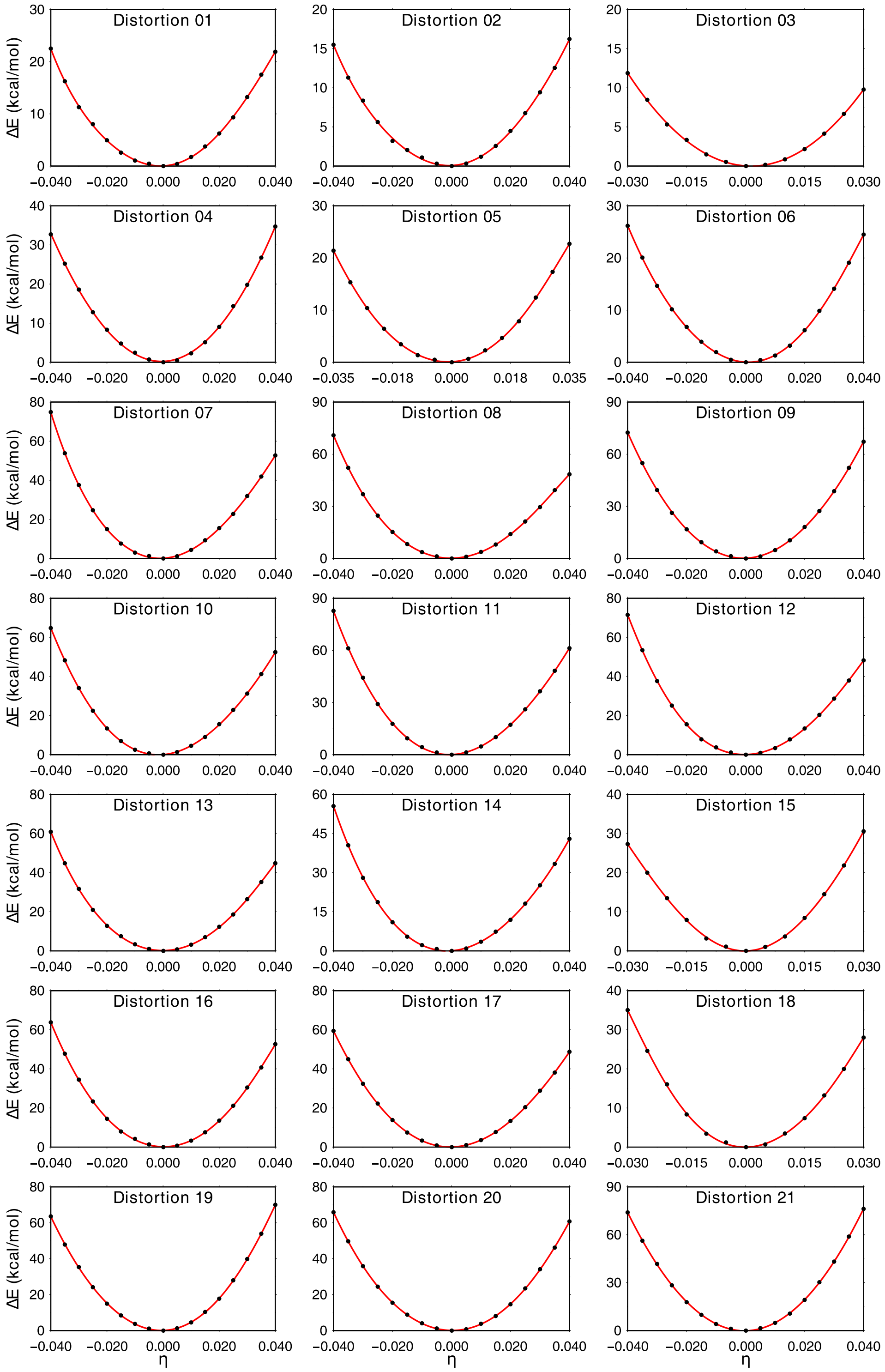

Supplementary Figure 20. Fitting curves for electronic energies for 21 independent distortions. A red solid line represents a polynomial fitting curve, while a black circle represents the electronic energy at a given distortion.

Supplementary Table 7. Calculated elastic moduli (GPa) and Poisson's ratio using the ElaStic program. (Voigt; V, Reuss; R)

**Bulk modulus**		**Young's modulus**		**Shear modulus**		**Poisson's ratio**	
B_V	B_R	E_V	E_R	G_V	G_R	v_V	v_R
8.52	6.25	11.04	7.84	4.30	3.04	0.28	0.29

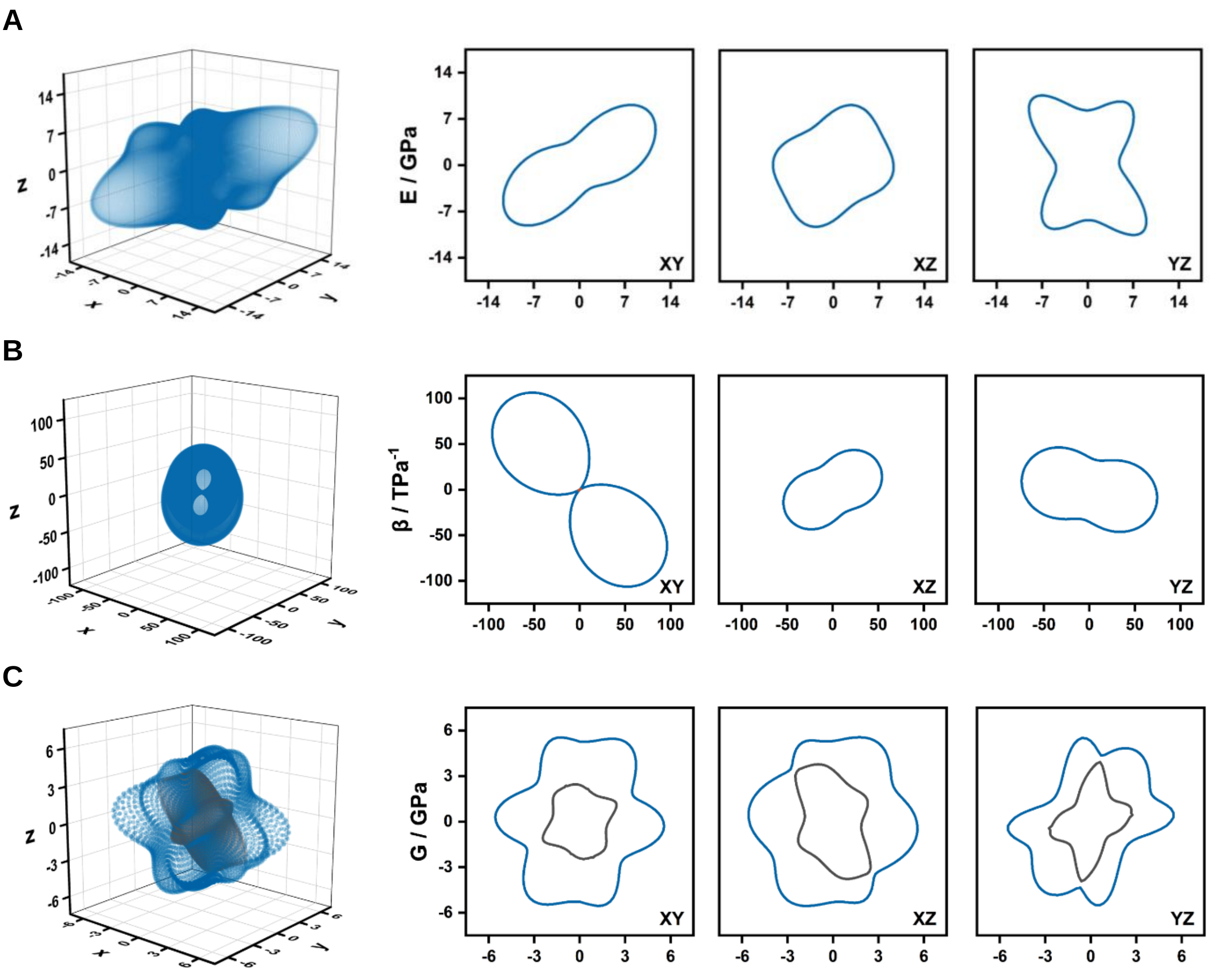

Supplementary Figure 21. 3D surfaces and 2D polar plots of elastic moduli for the xy, xz, and yz planes. **A** Young's modulus (E), **B** Linear compressibility (β), **C** Shear modulus (G). Blue and black lines represent the maximal and minimal positive values, respectively, while the red line represents the minimal negative values over all possible values.

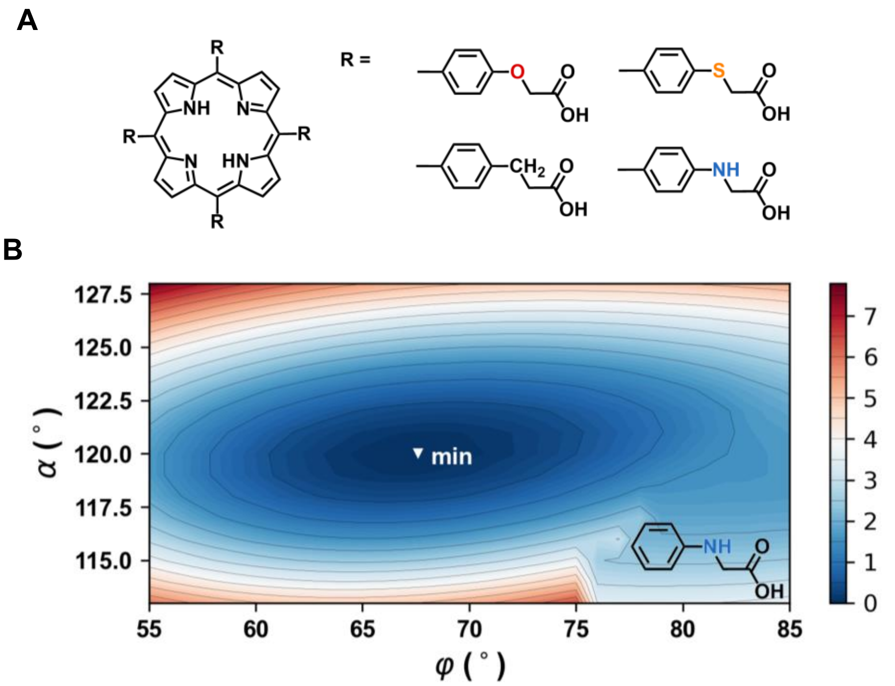

Supplementary Figure 22. Potential origamic MOFs by changing the functional groups of a porphyrinic linker. A The porphyrinic linker can be substituted by various functional groups; -O-, -CH_2-, -S-, and -NH-. **B** Potential energy surface of N-phenylglycine (-NH-) molecule by varying φ and α. This molecule can be stabilized in its more folded conformation than the aryloxy group.

Supplementary References

1. Karmakar, A. & Goldberg, I. Flexible porphyrin tetracarboxylic acids for crystal engineering. *CrystEngComm* **12**, 4095–4100 (2010).

2. Das, T., Chakraborty, S., Sarma, H. D., Banerjee, S. & Venakatesh, M. A novel ^{177}Lu-labeled porphyrin for possible use in targeted tumor therapy. *Nucl Med Biol*. **37**, 655–663 (2010).

3. Stephens, P. J., Devlin, F. J., Chabalowski, C. F. & Frisch, M. J. Ab Initio calculation of vibrational absorption and circular dichroism spectra using density functional force fields. *J. Phys. Chem.* **98**, 11623–11627 (1994).

4. Lee, C., Yang, W. & Parr, R. G. Development of the colle-salvetti correlation-energy formula into a functional of the electron density. *Phys. Rev. B* **37** (2), 785–789 (1988).

5. Becke, A. D. Density-functional exchange-energy approximation with correct asymptotic behavior. *Phys. Rev. A* **38** (6), 3098–3100 (1988).

6. Becke, A. D. Density-functional thermochemistry. III. The role of exact exchange. *J. Chem. Phys.* **98** (7), 5648–5652 (1993).

7. Frisch, M. J. et al. *Gaussian 16 Revision C.01*; Gaussian Inc.: Wallingford, CT, 2016.

8. Grimme, S., Ehrlich, S. & Goerigk, L. Effect of the damping function in dispersion corrected density functional theory. *J. Chem. Phys.* **32** (7), 1456–1465 (2011).

9. Dunning Jr., T. H. Gaussian basis sets for use in correlated molecular calculations. I. The atoms boron through neon and hydrogen. *J. Chem. Phys.* **90** (2), 1007–1023 (1989).

10. Kressen, G.; Furthmüller, J. Efficient iterative schemes for ab initio total-energy calculations using a plane-wave basis set. *Phys. Rev. B.* **54**, 11169–11186 (1996)

11. Perdew, J. P.; Burke, K.; Ernzerhof, M. Generalized Gradient Approximation Made Simple. *Phys. Rev. Lett.* **77**, 3865–3868 (**1997**).

12. Grimme, S.; Ehrlich, S.; Goerigk, L. Effect of the Damping Function in Dispersion Corrected Density Functional Theory. *J. Comput. Chem.* **32**, 1456–1465 (**2011**).

13. Golesorkhtabar, R., Pavone, P., Spitaler, J., Puschnig, P. & Draxl, C. ElaStic: A tool for calculating second-order elastic constants from first principles. *Comput. Phys. Commun.* **184**, 1861–1873 (2013).

14. Gaillac, R., Pullumbi, P. & Coudert, F.-X. ELATE: an open-source online application for analysis and visualization of elastic tensors. *J. Phys.: Condens. Matter* **28**, 275201 (2016).

15. Shi, Y.-X., Chen, H.-H., Zhang, W.-H., Day, G. S., Lang, J.-P. & Zhou, H.-C. Photoinduced nonlinear contraction behavior in metal-organic frameworks. *Chem. Eur. J.* **25**, 8543–8549 (2019).

16. Shrivastava, A. & Das, D. Axial positive, negative, and zero thermal expansion in a mixed-metal mixed-linker coordination compound: role of 2D layer in the thermal expansion property. *Cryst. Growth Des*. **19**, 4908–4913 (2019).

17. Zhou, H.-L., Zhang, J.-P. & Chen, X.-M. Controlling thermal expansion behaviors of fence-like metal-organic frameworks by varying/mixing metal ions. *Front. Chem*. **6**, 306 (2018).

18. Hibble, S. J., Chippindale, A. M., Pohl, A. H. & Hannon, A. C. Surprise from a simple material-The structure and properties of nickel cyanide. *Angew. Chem*. **119**, 7246–7248 (2007).

19. Yao, W. et al. Area negative thermal expansion in a beryllium borate $LiBeBO_3$ with edge sharing tetrahedral. *Chem. Commun*. **50**, 13499 (2014).
