# Supplementary material for: Origamic metal-organic framework toward mechanical metamaterial
Source: Nat Commun. 2023 Dec 1;14:7938. doi: 10.1038/s41467-023-43647-8 (PMC10692132; doi:10.1038/s41467-023-43647-8)
Supplement: Supplementary file 3 — Description of Additional Supplementary Files [file 41467_2023_43647_MOESM3_ESM.pdf]

Description of Additional Supplementary Files

File Name: Supplementary Data 1
Description: Crystallographic data of PPF-301 depending on temperatures from 100K to 380K at intervals of 20K.

File Name: Supplementary Movie 1
Description: A transformation animation of a regular DCS origami tessellation.
A regular DCS origami tessellation, consisting of same-sized squares, demonstrates a folding movement. This folding movement transforms a 2D plane into a 3D wave-like material. The folding angle theoretically ranges from 0° to 180°. The total area of unfolded plane is larger than that of the folded plane.

File Name: Supplementary Movie 2
Description: A folding motion of PPF-301 at variable temperatures.
The folding motion of PPF-301 was demonstrated using crystallographic data collected at temperatures ranging from 100 K to 380 K at 20 K intervals. To verify the origami movement, crystallographic structures were simplified to represent the DCS tessellation. The 2D porphyrinic layer of PPF-301 exhibits a folding movement depending on temperature.
