# Supplementary material for: Origamic metal-organic framework toward mechanical metamaterial
Source: Nat Commun. 2023 Dec 1;14:7938. doi: 10.1038/s41467-023-43647-8 (PMC10692132; doi:10.1038/s41467-023-43647-8)

checkCIF/PLATON report

Structure factors have been supplied for datablock(s) P-1_a

THIS REPORT IS FOR GUIDANCE ONLY. IF USED AS PART OF A REVIEW PROCEDURE FOR PUBLICATION, IT SHOULD NOT REPLACE THE EXPERTISE OF AN EXPERIENCED CRYSTALLOGRAPHIC REFEREE.

No syntax errors found. CIF dictionary Interpreting this report

Datablock: P-1_a

Bond precision: C-C = 0.0039 A Wavelength=0.63000

Cell:	a=10.921(2)	b=12.640(3)	c=14.041(3)
	alpha=100.74(3)	beta=101.16(3)	gamma=108.02(3)
Temperature:	180 K		

	Calculated	Reported
Volume	1744.2(8)	1744.1(7)
Space group	P -1	P -1
Hall group	-P 1	-P 1
Moiety formula	2(C30.89 H27.42 N3.63 O7.63 Zn1.50), 2.738(C3 H7 N O)	?
Sum formula	C70 H74 N10 O18 Zn3	C70 H74 N10 O18 Zn3
Mr	1539.56	1539.50
Dx,g cm-3	1.466	1.466
Z	1	1
Mu (mm-1)	0.796	0.796
F000	798.0	798.0
F000'	799.16	
h,k,lmax	15,17,19	15,17,19
Nref	10373	9745
Tmin,Tmax	0.828,0.904	0.547,0.906
Tmin'	0.504	

Correction method= # Reported T Limits: Tmin=0.547 Tmax=0.906
AbsCorr = EMPIRICAL

Data completeness= 0.939 Theta(max)= 26.497

R(reflections)= 0.0539(6670) wR2(reflections)= 0.1670(9745)

S = 1.028 Npar= 571

The following ALERTS were generated. Each ALERT has the format
test-name_ALERT_alert-type_alert-level.
Click on the hyperlinks for more details of the test.

Alert level B

```
PLAT029_ALERT_3_B _diffrn_measured_fraction_theta_full value Low .      0.956 Why?
```

Author Response: Since the Pohang Accelerator Laboratory 2D Beamline goniostat has only one omega-axis, diffrn_measured_fraction_theta_full is not fully covered as 0.956, especially for the low symmetry such as triclinic system. As this is an inherent problem, other command and option were not helpful to improve the completeness.

Alert level C

```
PLAT213_ALERT_2_C Atom C5B             has ADP max/min Ratio .....         3.6 oblate
PLAT220_ALERT_2_C NonSolvent   Resd 1  C   Ueq(max)/Ueq(min) Range         3.7 Ratio
PLAT260_ALERT_2_C Large Average Ueq of Residue Including       O3B      0.105 Check
PLAT906_ALERT_3_C Large K Value in the Analysis of Variance ......      2.214 Check
PLAT911_ALERT_3_C Missing FCF Refl Between Thmin & STh/L=    0.600        275 Report
PLAT977_ALERT_2_C Check Negative Difference Density on H7A              -0.34 eA-3
```

Alert level G

```
ABSMU01_ALERT_1_G  Calculation of _exptl_absorpt_correction_mu
                  not performed for this radiation type.
PLAT002_ALERT_2_G Number of Distance or Angle Restraints on AtSite          30 Note
PLAT003_ALERT_2_G Number of Uiso or Uij Restrained non-H Atoms ...          15 Report
PLAT004_ALERT_5_G Polymeric Structure Found with Maximum Dimension           2 Info
PLAT063_ALERT_4_G Crystal Size Possibly too Large for Beam Size ..        0.86 mm
PLAT092_ALERT_4_G Check: Wavelength Given is not Cu,Ga,Mo,Ag,In Ka    0.63000 Ang.
PLAT154_ALERT_1_G The s.u.'s on the Cell Angles are Equal ..(Note)        0.03 Degree
PLAT168_ALERT_4_G The CIF-Embedded .res File Contains EXYZ Records           3 Report
PLAT171_ALERT_4_G The CIF-Embedded .res File Contains EADP Records           3 Report
PLAT172_ALERT_4_G The CIF-Embedded .res File Contains DFIX Records          18 Report
PLAT173_ALERT_4_G The CIF-Embedded .res File Contains DANG Records          18 Report
PLAT174_ALERT_4_G The CIF-Embedded .res File Contains FLAT Records           6 Report
PLAT177_ALERT_4_G The CIF-Embedded .res File Contains DELU Records           3 Report
PLAT178_ALERT_4_G The CIF-Embedded .res File Contains SIMU Records           3 Report
PLAT186_ALERT_4_G The CIF-Embedded .res File Contains ISOR Records           2 Report
PLAT187_ALERT_4_G The CIF-Embedded .res File Contains RIGU Records           6 Report
PLAT300_ALERT_4_G Atom Site Occupancy of Zn2        Constrained at         0.5 Check
PLAT301_ALERT_3_G Main Residue  Disorder ..............(Resd  1  )         20% Note
PLAT302_ALERT_4_G Anion/Solvent/Minor-Residue Disorder (Resd  2  )        100% Note
PLAT302_ALERT_4_G Anion/Solvent/Minor-Residue Disorder (Resd  3  )        100% Note
PLAT302_ALERT_4_G Anion/Solvent/Minor-Residue Disorder (Resd  4  )        100% Note
PLAT413_ALERT_2_G Short Inter XH3 .. XHn     H25B      ..H2B3     .       2.10 Ang.
                                                1-x,1-y,1-z  =      2_666 Check
PLAT720_ALERT_4_G Number of Unusual/Non-Standard Labels ..........          36 Note
PLAT779_ALERT_4_G Suspect or Irrelevant (Bond) Angle(s) in CIF ...       17.26 Deg.
               ZN2  -N1   -ZN2     1_555   1_555   2_766 ......     #   43 Check
PLAT779_ALERT_4_G Suspect or Irrelevant (Bond) Angle(s) in CIF ...       17.57 Deg.
```

```
            ZN2  -N2   -ZN2     1_555   1_555   2_766 ......     #   49 Check
PLAT804_ALERT_5_G Number of ARU-Code Packing Problem(s) in PLATON          1 Info
PLAT811_ALERT_5_G No ADDSYM Analysis: Too Many Excluded Atoms ....         ! Info
PLAT860_ALERT_3_G Number of Least-Squares Restraints ............        312 Note
PLAT883_ALERT_1_G No Info/Value for _atom_sites_solution_primary .     Please Do !
PLAT912_ALERT_4_G Missing # of FCF Reflections Above STh/L=  0.600       354 Note
PLAT941_ALERT_3_G Average HKL Measurement Multiplicity ..........        2.0 Low
PLAT978_ALERT_2_G Number C-C Bonds with Positive Residual Density.         6 Info
PLAT984_ALERT_1_G The Zn-f'=    0.3273 Deviates from the B&C-Value     0.3261 Check
PLAT985_ALERT_1_G The Zn-f"=    1.1646 Deviates from the B&C-Value     1.1614 Check
```

```
  0 ALERT level A = Most likely a serious problem - resolve or explain
  1 ALERT level B = A potentially serious problem, consider carefully
  6 ALERT level C = Check. Ensure it is not caused by an omission or oversight
 34 ALERT level G = General information/check it is not something unexpected

  5 ALERT type 1 CIF construction/syntax error, inconsistent or missing data
  8 ALERT type 2 Indicator that the structure model may be wrong or deficient
  6 ALERT type 3 Indicator that the structure quality may be low
 19 ALERT type 4 Improvement, methodology, query or suggestion
  3 ALERT type 5 Informative message, check
```

It is advisable to attempt to resolve as many as possible of the alerts in all categories. Often the minor alerts point to easily fixed oversights, errors and omissions in your CIF or refinement strategy, so attention to these fine details can be worthwhile. In order to resolve some of the more serious problems it may be necessary to carry out additional measurements or structure refinements. However, the purpose of your study may justify the reported deviations and the more serious of these should normally be commented upon in the discussion or experimental section of a paper or in the "special_details" fields of the CIF. checkCIF was carefully designed to identify outliers and unusual parameters, but every test has its limitations and alerts that are not important in a particular case may appear. Conversely, the absence of alerts does not guarantee there are no aspects of the results needing attention. It is up to the individual to critically assess their own results and, if necessary, seek expert advice.

Publication of your CIF in IUCr journals

A basic structural check has been run on your CIF. These basic checks will be run on all CIFs submitted for publication in IUCr journals (*Acta Crystallographica*, *Journal of Applied Crystallography*, *Journal of Synchrotron Radiation*); however, if you intend to submit to *Acta Crystallographica Section C* or *E* or *IUCrData*, you should make sure that full publication checks are run on the final version of your CIF prior to submission.

Publication of your CIF in other journals

Please refer to the *Notes for Authors* of the relevant journal for any special instructions relating to CIF submission.

PLATON version of 13/07/2021; check.def file version of 13/07/2021

Datablock P-1_a - ellipsoid plot

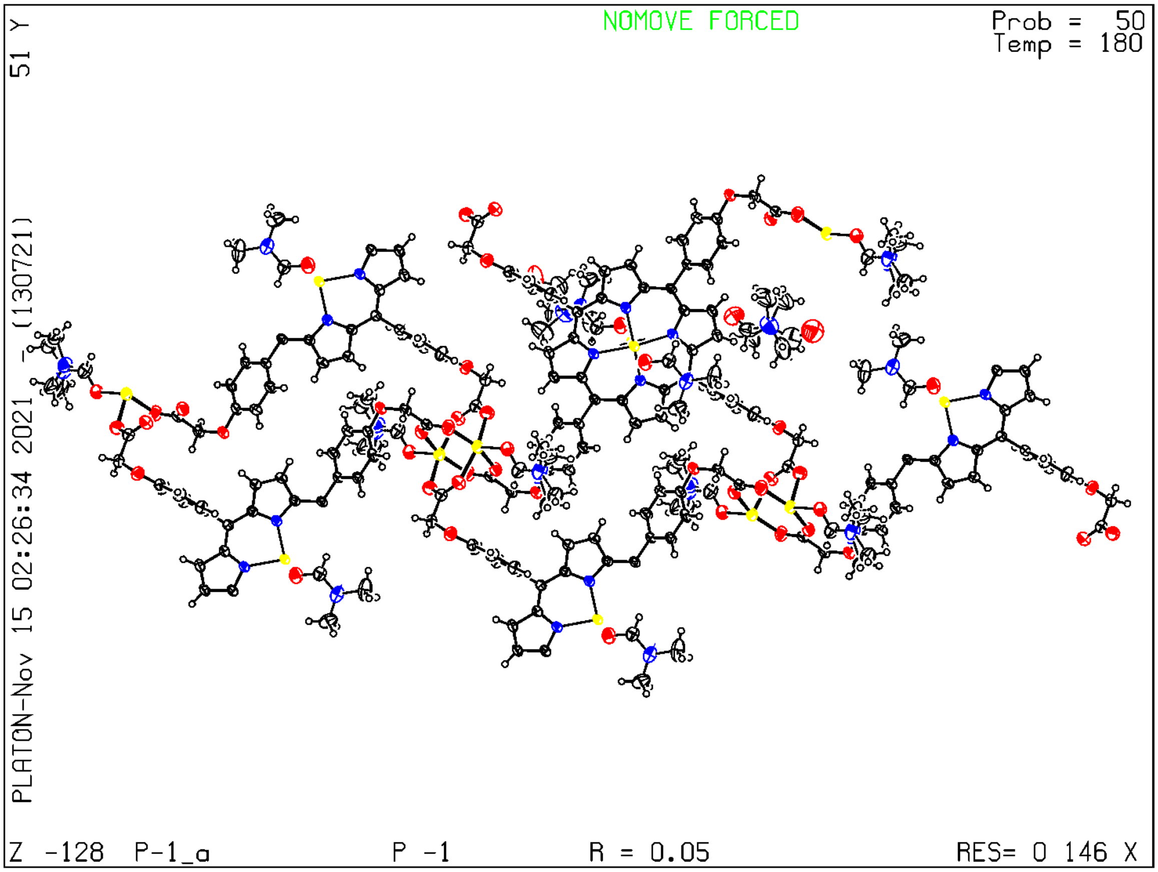

Supplement: Supplementary file 4 — Supplementary Data 1 [file checkcif_180K.pdf › Crystallographic data/checkcif_180K.pdf]
